# Long-term efficacy and safety of perampanel in patients aged 60 years and older with focal seizures: Post hoc analysis of phase III open-label extension studies stratified by enzyme-inducing anti-seizure medication use

**DOI:** 10.1016/j.ebr.2025.100833

**Published:** 2025-10-10

**Authors:** Rohit Marawar, Ilo E. Leppik, Robert T. Wechsler, Anna Patten, Leock Y. Ngo

**Affiliations:** a Wayne State University, Detroit, MI, USA; b University of Minnesota, Minneapolis, MN, USA; c Idaho Comprehensive Epilepsy Center, Boise, ID, USA; d Eisai Europe Ltd., Hatfield, Hertfordshire, UK; e Eisai Inc., Nutley, NJ, USA

**Keywords:** Epilepsy, Adjunctive therapy, Perampanel, Focal to bilateral tonic-clonic seizures, Older adults

## Abstract

Examine long-term findings from two Phase III open-label extension (OLEx) studies of adjunctive perampanel in patients aged ≥60 years experiencing focal seizures (FS), with/without focal to bilateral tonic-clonic seizures (FBTCS), with/without enzyme-inducing anti-seizure medications (EIASMs). Methods: Study 307 (NCT00735397) included a Conversion Period (16 weeks; blinded) and a Maintenance Period (256 weeks). Study 335 OLEx (NCT01618695) included Pre-conversion (4 weeks), Conversion (6 weeks), and Maintenance Periods (≥46 weeks). Perampanel (2–12 mg/day) was given alongside 1–3 concomitant ASMs, of which ≤2 (Study 307) and ≤1 (Study 335) were EIASMs. Efficacy and safety outcomes were analyzed. Results: Seventy-one patients aged ≥60 years were included in the Full and Safety Analysis Sets. Over 4 years, seizure frequency decreased regardless of EIASM use; seizure-freedom rates for FS were 0.0 % (n = 0/71) for Year 1, 2.6 % (n = 1/38) Year 2, 5.3 % (n = 1/19) Year 3, and 0.0 % (n = 0/14) Year 4. For FBTCS, these rates were 26.3 % (n = 5/19) for Year 1, 22.2 % (n = 2/9) Year 2, 40.0 % (n = 2/5) Year 3, and 0.0 % (n = 0/4) Year 4. The 90 % responder rates were ≥14.0 % for FS and ≥40.0 % for FBTCS. Incidence of treatment-emergent adverse events (TEAEs) was highest during Year 1 (87.3 % [n = 62/71]) but decreased across Years 2–4 (47.4 %–60.4 %). The most commonly reported TEAE during Years 1/2 was dizziness (47.9 % [n = 34/71] and 12.5 % [n = 6/48], respectively) and during Years 3/4 was fall (15.8 % [n = 3/19] and 14.3 % [n = 2/14]). Significance: Adjunctive perampanel conferred long-term seizure control in older patients with epilepsy; safety was aligned with established perampanel safety profile.

## Highlights

• Adjunctive perampanel conferred long-term seizure control in older patients.
• During Year 1, older adults had a 95.5 % median percent reduction in FBTCS frequency.
• The most common TEAEs were dizziness (Years 1/2) and fall (Years 3/4).
• Adjunctive perampanel long-term safety was aligned with established safety profile.

## 1 Introduction

Epilepsy is estimated to impact 50 million people across the globe, and is among the most prevalent neurological conditions [1,2]. Prevalence is high in older adults (aged ≥65 years; 1–1.5 %), especially among nursing home residents where the prevalence of epilepsy can exceed 5 % [3,4]. The average age of the global population is increasing, and the number of older adults who develop epilepsy will continue to increase worldwide [5,6]. However, participation by older adult patients in clinical studies of anti-seizure medications (ASMs) is often limited because of the potential for age-related comorbidities to interfere with study participation and affect clinical outcomes [7,8]. This has resulted in a paucity of clinical data on ASM use that could guide treatment decisions in older adults [8,9]. Hence, evaluating ASMs in older adult patients with epilepsy enrolled in clinical trials is essential.

In a sub-analysis of three Phase III studies (Study 304, NCT00699972; Study 305, NCT00699582; Study 306, NCT00700310), perampanel was reported to have comparable efficacy in older (aged 65 years and older) and younger adult patients (aged 18 to less than 65 years), with similar incidences of treatment-emergent adverse events (TEAEs) in both age groups [9]. However, older adult patients experienced falls, dizziness, and fatigue more commonly than younger adult patients [9]. Pharmacokinetic data suggested that enzyme-inducing anti-seizure medications (EIASMs) increase clearance of perampanel; thus, patients treated with EIASMs and perampanel concomitantly may need a higher dose of perampanel to attain similar efficacy as patients treated with non-EIASMs [[10], [11], [12]].

Studies 307 (NCT00735397) and 335 open-label extension (OLEx; NCT01618695), two OLEx studies, evaluated perampanel long-term clinical outcomes in patients with focal seizures (FS), with or without focal to bilateral tonic-clonic seizures (FBTCS) [13,14]. To build on previous findings of perampanel use in older adults [9,[15], [16], [17]], we conducted a post hoc analysis in a subgroup of patients aged ≥60 years enrolled in Studies 307 and 335 OLEx. Additionally, we evaluated perampanel efficacy and safety in older patients receiving concomitant EIASMs (defined as carbamazepine, eslicarbazepine, phenytoin, oxcarbazepine) as well as those receiving non-EIASMs.

## 2 Methods

### 2.1 Study designs

Detailed methods for Studies 307 and 335 OLEx have been published [13,14]. In brief, Study 307 (NCT00735397) enrolled patients with inadequately controlled FS, including FBTCS, who had completed one of the following double-blind Phase III studies: Study 304 (NCT00699972), Study 305 (NCT00699582), or Study 306 (NCT00700310) [13]; Study 307 included a Conversion Period (16 weeks; blinded) and a Maintenance Period (≥256 weeks; open-label) [13]. Study 335 OLEx (NCT01618695) enrolled patients who had previously completed the Core Study 335. The Study 335 OLEx Phase comprised a Pre-conversion Period (4 weeks), a Conversion Period (6 weeks), and a Maintenance Period (≥46 weeks) [14]. Patients received 1–3 concomitant ASMs in Studies 307 and 335, of which ≤2 and 1 ASMs, respectively, were permitted to be EIASMs such as carbamazepine, eslicarbazepine, oxcarbazepine, phenobarbital, and phenytoin. In Study 307, investigators had the discretion to modify the number and dose of concomitant ASMs. In Study 335 OLEx, the concomitant ASM and its dose were to remain stable when the perampanel dose was being titrated unless emergency care was needed. Otherwise, these parameters were permitted to change at the discretion of the investigator.

In Study 307 and Study 335 OLEx, all patients who were randomized to placebo during the lead-in studies were switched to perampanel 2 mg/day during the Conversion Period and were up-titrated biweekly (Study 307) or weekly (Study 335 OLEx) in 2-mg increments until reaching an optimal dose of perampanel that did not exceed 12 mg/day. Titrations were based on individual tolerance, the investigator’s clinical judgment, and the patient’s willingness to increase the perampanel dose. Additionally, all patients who received less than 12 mg/day of perampanel during the lead-in studies had their dose titrated up to 12 mg/day (or an optimal dose based on tolerability). During the OLEx Maintenance Periods, the study treatment was disclosed to all patients and they continued to take their optimal perampanel dose that was determined during the Conversion Period. However, during the OLEx Maintenance Period of both studies, investigators were allowed to adjust perampanel dose if clinically required. Patients discontinued the study if they were unable to tolerate perampanel at a dose of 2 mg/day.

Patient disposition and primary reasons for discontinuation in Study 307 were reported previously [13]. Patients continued in the study unless they withdrew because of the study closure, commercial unavailability of perampanel in their country, adverse events, patient choice, or inadequate therapeutic effect. The duration of the OLEx Maintenance Period in Study 335 OLEx was ≥46 weeks up to 75 weeks; however, the OLEx Maintenance Period was terminated within 3 months in countries where perampanel received marketing approval prior to Week 75. Written informed consent was obtained from all patients prior to their participation. The protocols for Studies 307 and Study 335 OLEx underwent review by national regulatory authorities in conjunction with institutional review boards and independent ethics committees [13,14].

### 2.2 Post hoc analyses

#### 2.2.1 Post hoc analysis in older adults

Adjunctive perampanel efficacy and safety outcomes in older adult patients with FS, with or without FBTCS, were evaluated post hoc. Clinical data from patients who were 60 years or older at the time of informed consent from Studies 307 and 335 OLEx were pooled in Years 1–4; Year 1 Day 1 corresponded with the date of first dose of perampanel. The duration of perampanel treatment started from the first dose in the double-blind or open-label study to the last dose in the open-label study; however, for patients with a >14-day gap in perampanel exposure from the double-blind to the open-label study, treatment duration started from the first dose of perampanel in the open-label study.

Efficacy assessments by seizure counts were split by 1-year treatment intervals and were carried out in the Full Analysis Set (FAS). All patients who received at least 1 dose of perampanel in Studies 307 (and/or Core Study) or 335 OLEx and had valid seizure data during perampanel treatment were included in the FAS. Efficacy assessments included median percent reduction in seizure frequency per 28 days compared with baseline (baseline was defined as the Pre-randomization Period during the lead-in studies); 50 %, 75 %, and 90 % responder rates (defined as ≥50 %, ≥75 %, and ≥90 % seizure frequency reduction, respectively); seizure-freedom rates (seizure counts were calculated using seizure diaries).

Safety assessments were calculated in the Safety Analysis Set (SAS), which comprised all patients who received at least 1 dose of perampanel and had at least 1 safety assessment after receiving a treatment dose in Studies 307 or 335 OLEx. These assessments included monitoring of TEAEs, serious TEAEs, and withdrawal as a result of TEAEs.

#### 2.2.2 Post hoc analysis by baseline concomitant EIASM use

In the sub-analysis by EIASM use, the efficacy and safety data from patients aged ≥60 years with FS, including FBTCS, were pooled from Studies 307 and 335 OLEx and stratified by concomitant EIASM use at baseline over Years 1–4. Efficacy assessments, split by 52-week treatment intervals, were based on the FAS from Studies 307 and 335 OLEx. The FAS included patients who gave informed consent, received at least 1 dose of study drug, and had any efficacy data following dosing during treatment duration. Assessments comprised of median percent reduction in seizure frequency per 28 days relative to baseline; 50 %, 75 %, and 90 % responder rates; and seizure-freedom rates. Safety assessments included monitoring of TEAEs and were based on the SAS.

## 3 Results

### 3.1 Patients

Overall, 71 patients aged ≥60 years with FS were included in the FAS, of whom 42 had FS with FBTCS. Prior to unblinding, 50 patients were randomized to perampanel and 21 to placebo (Table S1 details patient disposition for this post hoc analysis). Table 1 includes a summary of patients’ baseline demographics and clinical characteristics stratified by baseline EIASM status. Of the 71 patients, 70 (98.6 %) were aged ≥60 to <75 years, and at baseline, the majority (88.7 % [n = 63/71]) were treated with ≥2 concomitant ASMs. Additionally, more patients (62.0 % [n = 44/71]) were receiving EIASMs than non-EIASMs (38.0 % [n = 27/71]) at baseline.

**Table 1:** Demographic information and baseline^a^ characteristics of older adult patients, stratified by concomitant EIASM use recorded at baseline (Safety Analysis Set).

	**EIASMs**^b^ **(n = 44)**	**Non-EIASMs** **(n = 27)**	**All patients** **(N = 71)**
**Median (min, max) age,**^c^**years**	63 (60, 76)	63 (60, 69)	63.0 (60, 76)
**Age group, n (%)**			
≥60 to <75 years	43 (97.7)	27 (100.0)	70 (98.6)
≥75 years	1 (2.3)	0 (0.0)	1 (1.4)
**Female, n (%)**	27 (61.4)	16 (59.3)	43 (60.6)
**Race, n (%)**			
Black or African American	1 (2.3)	1 (3.7)	2 (2.8)
Chinese	5 (11.4)	2 (7.4)	7 (9.9)
Japanese	7 (15.9)	3 (11.1)	10 (14.1)
White	25 (56.8)	18 (66.7)	43 (60.6)
Other Asian	6 (13.6)	3 (11.1)	9 (12.7)
**Type of seizure, n (%)**			
Focal preserved consciousness without motor phenomenon	16 (36.4)	10 (37.0)	26 (36.6)
Focal preserved consciousness with motor phenomenon	8 (18.2)	8 (29.6)	16 (22.5)
Focal impaired consciousness	41 (93.2)	22 (81.5)	63 (88.7)
FS with FBTCS	27 (61.4)	15 (55.6)	42 (59.2)
**Number of ASMs recorded at baseline,**^d^**n (%)**			
1	5 (11.4)	3 (11.1)	8 (11.3)
2	19 (43.2)	14 (51.9)	33 (46.5)
3	20 (45.5)	10 (37.0)	30 (42.3)
**Most common baseline ASMs (in ≥20.0 % of total patients), n (%)**			
**EIASMs**	44 (100.0)	0 (0.0)	44 (62.0)
Carbamazepine	22 (50.0)	0 (0.0)	22 (31.0)
Oxcarbazepine	15 (34.1)	0 (0.0)	15 (21.1)
**Non-EIASMs**	36 (81.8)	27 (100.0)	63 (88.7)
Levetiracetam	10 (27.8)	16 (59.3)	26 (36.6)
Lamotrigine	10 (27.8)	9 (33.3)	19 (26.8)
Valproic acid	6 (16.7)	12 (44.4)	18 (25.4)

Percentages are calculated based on the total number of patients with non-missing values in the applicable treatment group.

ASMs, anti-seizure medications; EIASMs, enzyme-inducing anti-seizure medications; FBTCS, focal to bilateral tonic-clonic seizures; FS, focal seizures; max, maximum; min, minimum.

a Baseline is defined as the Pre-randomization Period during the Double-blind Phase.

b EIASMs are defined as carbamazepine, eslicarbazepine, phenytoin, and oxcarbazepine. All other ASMs are non-EIASMs.

c Age was determined based on the date of informed consent.

d Patients who reported the same ASM more than once are counted only once.

The mean modal (standard deviation; SD) dose of perampanel for the overall population across Years 1–4 was 8.9 (3.2) mg/day (Table S2). The two most common modal doses received by all patients throughout Studies 307 and 335 OLEx were 12 mg/day (39.4 % [n = 28/71]) and 8 mg/day (23.9 % [n = 17/71]). For the subsets of patients treated with or without EIASMs, the mean modal (SD) dose of perampanel was 8.9 (3.5) mg/day and 8.9 (2.6) mg/day, respectively (Table S2). The most common modal doses received by patients in this sub-analysis across Years 1–4 were 12 mg/day (EIASMs, 45.5 % [n = 20/44]; non-EIASMs, 29.6 % [n = 8/27]) and 8 mg/day (EIASMs, 15.9 % [n = 7/44]; non-EIASMs, 37.0 % [n = 10/27]). At the end of Studies 307 and 335 OLEx, three patients (4.2 % [n = 3/44]) switched to non-EIASMs after receiving EIASMs at baseline; no switches to EIASMs were reported in patients who reported receiving non-EIASMs at baseline.

### 3.2 Efficacy outcomes

#### 3.2.1 Percent reduction in seizure frequency per 28 days

**Fig. 1:**
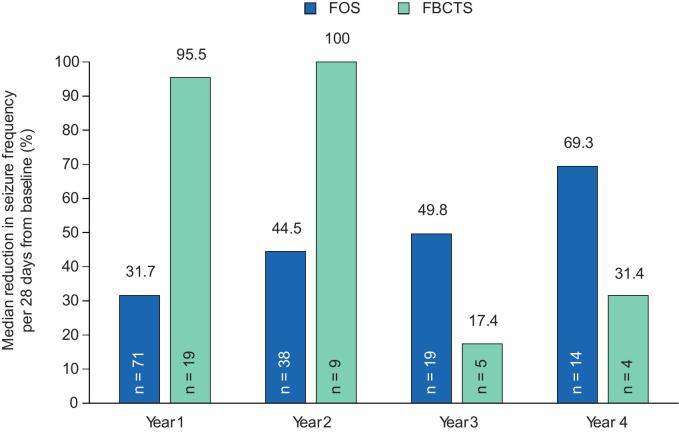

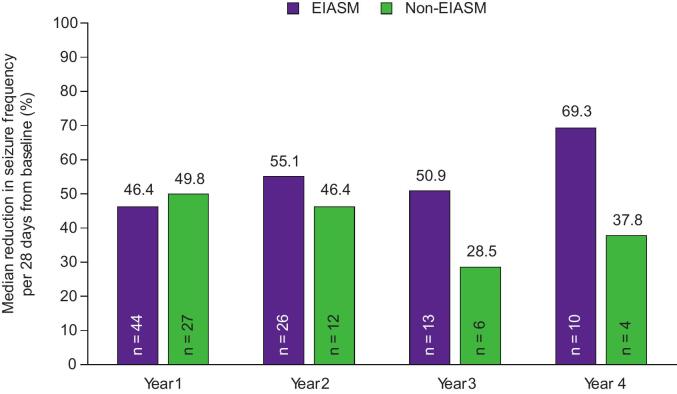
A) Median percent reduction in seizure frequency per 28 days from baseline over 4 years in patients aged ≥60 years, and B) median percent reduction in seizure frequency per 28 days from baseline over 4 years in patients aged ≥60 years, stratified by concomitant EIASM use recorded at baseline (Full Analysis Set). EIASM, enzyme-inducing anti-seizure medication; FBTCS, focal to bilateral tonic-clonic seizure; FS, focal seizure.

Median percent reductions in seizure frequency from baseline for over 4 years with adjunctive perampanel are in Fig. 1A. Patients with FS (n = 71) experienced a median percent reduction in total seizure frequency of 31.7 % in Year 1. While reductions in total seizure frequency were greater in Years 2–4 (44.5 %–69.3 %), the number of patients with FS available for analysis declined over time. In the subset of patients with FBTCS (n = 19), the median percent reduction in FBTCS frequency was 95.5 % during Year 1 and was widely variable during Years 2–4 (17.4 %–100.0 %); however, the number of patients with FBTCS available for analysis during Years 2–4 was small. Reductions in seizure frequency from baseline stratified by concomitant EIASM use in patients aged ≥60 years were also observed over 4 years following initiation of perampanel treatment (Fig. 1B). During Year 1, percent reductions in seizure frequency were comparable in the EIASM and the non-EIASM subgroups (46.4 % vs 49.8 %, respectively). During Years 2, 3, and 4, the observed percent reductions in seizure frequency were greater in patients receiving concomitant EIASMs at baseline than those receiving non-EIASMs (50.9–69.3 % vs 28.5–46.4 %, respectively); however, small patient numbers in Years 2–4, especially of those receiving non-EIASMs, may limit a meaningful comparison between these subgroups for these time periods.

#### 3.2.2 Responder, seizure-freedom, and retention rates

**Fig. 2:**
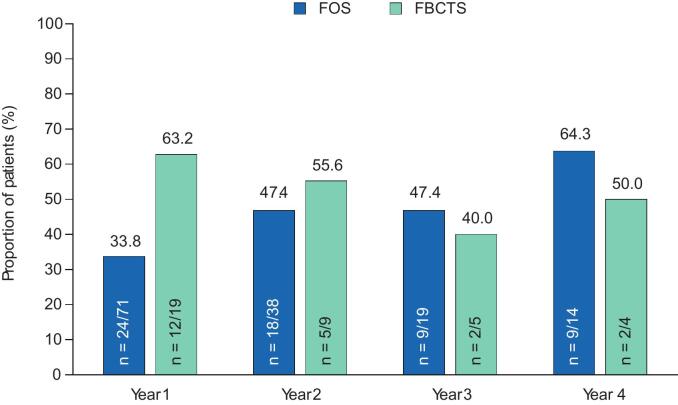

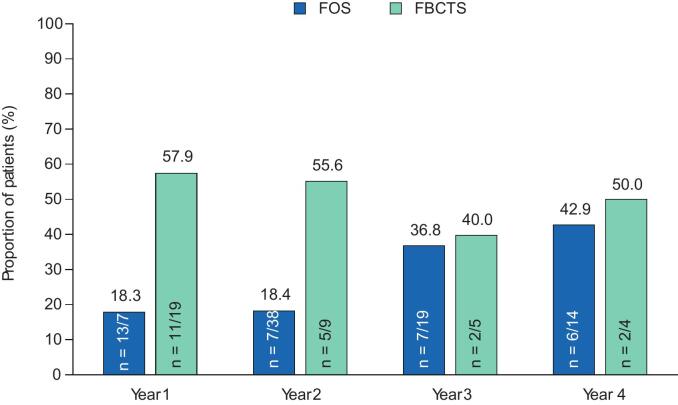

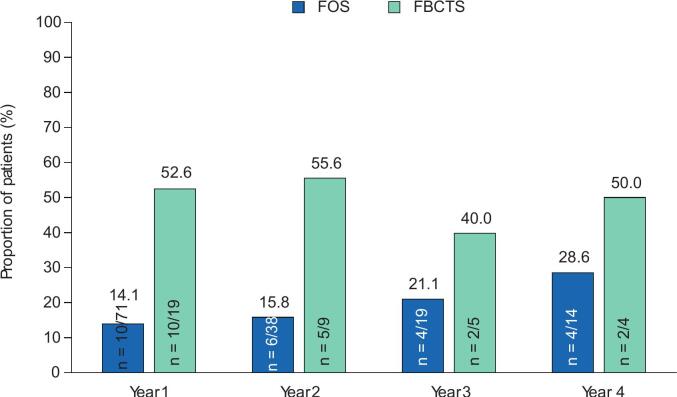

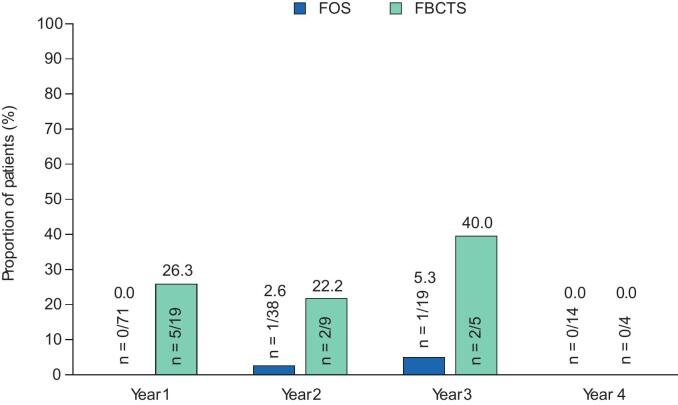
A) 50 % responder rates, B) 75 % responder rates, C) 90 % responder rates, and D) seizure-freedom rates over 4 years in patients aged ≥60 years (Full Analysis Set). FBTCS, focal to bilateral tonic-clonic seizure; FS, focal seizure.

The responder rates with adjunctive perampanel at Years 1–4 are presented in Fig. 2A–C. During Year 1, the 50 % responder rate in patients with FS was 33.8 % (n = 24/71), whereas the 75 % and 90 % responder rates were 18.3 % (n = 13/71) and 14.1 % (n = 10/71), respectively. In the subset of patients with FBTCS, the 50 % responder rate was 63.2 % (n = 12/19), whereas the 75 % and 90 % responder rates were 57.9 % (n = 11/19) and 52.6 % (n = 10/19), respectively. During Years 2–4, the 50 %, 75 %, and 90 % responder rates in patients with FS remained similar to Year 1 rates or increased. Among patients with FBTCS, corresponding responder rates during Years 2–4 were variable, with no particular trend noted in the setting of small sample sizes. In patients with FS, no seizure freedom was reported in any patient during Year 1 (Fig. 2D); seizure-freedom rates were 2.6 % (n = 1/38) during Year 2, 5.3 % (n = 1/19) during Year 3, and 0.0 % (n = 0/14) during Year 4. In the subset of patients with FBTCS, 26.3 % (n = 5/19) reported freedom from FBTCS seizures during Year 1 and 22.2 % (n = 2/9), 40.0 % (n = 2/5), and 0.0 % (n = 0/4) reported freedom from FBTCS seizures during Years 2, 3, and 4, respectively.

**Fig. 3:**
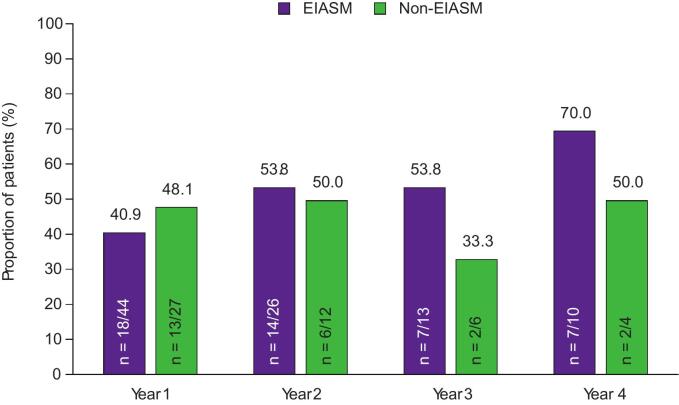

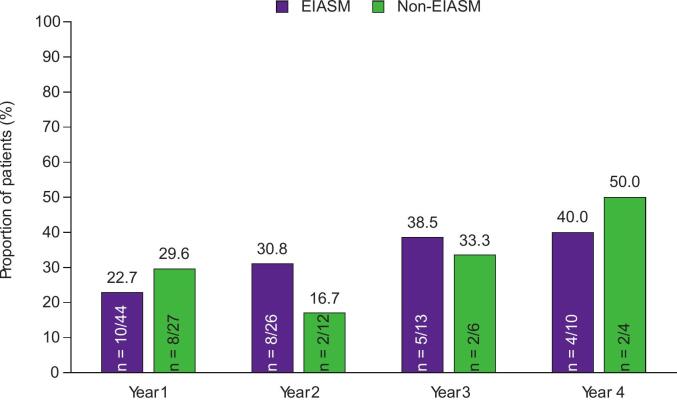

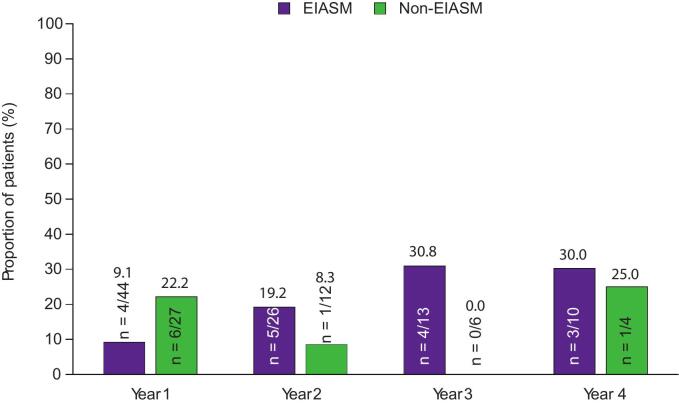

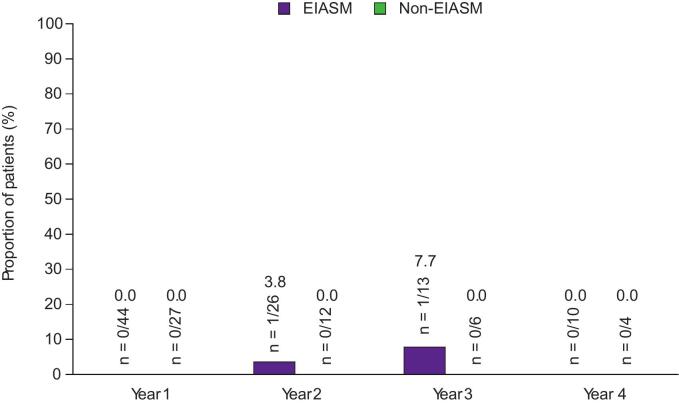
A) 50 % responder rates, B) 75 % responder rates, C) 90 % responder rates, and D) seizure-freedom rates over 4 years in patients aged ≥60 years, stratified by concomitant EIASM use recorded at baseline (Full Analysis Set). EIASM, enzyme-inducing anti-seizure medication.

The responder rates with adjunctive perampanel by concomitant EIASM use are shown in Fig. 3A–C. While responder rates during Year 1 were comparable between patients treated with EIASMs and patients treated with non-EIASMs, there were greater variations in responder rates between the two subgroups during later years.

During Year 1, the 50 % responder rate was comparable between patients treated with EIASMs and those treated with non-EIASMs (40.9 % vs 48.1 %, respectively). In the EIASM group, the 50 % responder rate was the same in Year 2 and 3 (53.8 %), though it increased to 70.0 % in Year 4. In the non-EIASM subgroup, the 50 % responder rates in Years 2, 3, and 4 were ≥33.3 %. The 75 % and the 90 % responder rates in patients treated with EIASMs during Year 1 were 22.7 % and 9.1 %, respectively, compared with 29.6 % and 22.2 % of those receiving non-EIASMs. During Years 2, 3, and 4, the 75 % responder rates were ≤40.0 % in the EIASM subgroup, whereas the 90 % responder rates were ≤30.8 %. In the non-EIASM group, the 75 % responder rates were ≤50.0 % in Years 2, 3, and 4, whereas the 90 % responder rates in this group were ≤25.0 %.

Seizure-freedom rates in patients receiving EIASMs or non-EIASMs are presented in Fig. 3D. One patient in the EIASM subgroup achieved seizure freedom during Years 2 and 3, whereas no patients achieved seizure freedom during Years 1 and 4; no patients achieved seizure-freedom in the non-EIASM group during any of the years.

Overall, the retention rates in patients aged ≥60 years with FS were 67.6 % (n = 48/71) at Year 1, 26.8 % (n = 19/71) at Year 2, and 19.7 % (n = 14/71) at Year 3. By Year 4, no patients were still receiving perampanel within the study. However, 12 patients continued perampanel treatment after study discontinuation through an expanded access program or via commercial supply, though those who switched to commercial supply were not counted as ongoing. Furthermore, not all patients were followed for the full 4-year study duration. An analysis of time to discontinuation over 4 years in patients aged ≥60 years is presented in Fig. S1.

### 3.3 Safety outcomes

Overall, the SAS included 71 patients aged ≥60 years; a total of 63.4 % of patients (n = 45/71) experienced an adverse event (AE) that led to a dose reduction. Notably, none of these patients discontinued treatment as a result of the AE; all continued on perampanel following the dose adjustment. The incidences of TEAEs in older adult patients were reported in 87.3 % (n = 62/71) of patients during Year 1, 60.4 % (n = 29/48) of patients during Year 2, 47.4 % (n = 9/19) of patients during Year 3, and 57.1 % (n = 8/14) of patients during Year 4 (Table 2). The most frequently reported TEAEs during Year 1 were dizziness (47.9 % [n = 34/71]) and somnolence (21.1 % [n = 15/71]) while during Year 2, these were dizziness (12.5 % [n = 6/48]) and laceration (10.4 % [n = 5/48]). Fall was the most frequently reported TEAE during Years 3 and 4 (15.8 % [n = 3/19] and 14.3 % [n = 2/14], respectively). The incidences of treatment-related TEAEs in patients aged ≥60 years were 84.5 % (n = 60/71) during Year 1, 31.3 % (n = 15/48) during Year 2, 21.1 % (n = 4/19) during Year 3, and 21.4 % (n = 3/14) during Year 4. Serious TEAEs occurred in 22.5 % (n = 16/71) of patients during Year 1, 20.8 % (n = 10/48) of patients during Year 2, 5.3 % (n = 1/19) of patients during Year 3, and 7.1 % (n = 1/14) of patients during Year 4. One death (73-year-old male) due to chronic obstructive pulmonary disease was recorded during Year 2; however, the death was deemed not perampanel-related per the investigator’s judgment.

**Table 2:** Overview of safety outcomes over 4 years in older adult patients (Safety Analysis Set).

	**Year 1** **(n = 71)**	**Year 2** **(n = 48)**	**Year 3** **(n = 19)**	**Year 4** **(n = 14)**
**TEAEs, n (%)**	62 (87.3)	29 (60.4)	9 (47.4)	8 (57.1)
**Treatment-related TEAEs, n (%)**	60 (84.5)	15 (31.3)	4 (21.1)	3 (21.4)
**Serious TEAEs, n (%)**	16 (22.5)	10 (20.8)	1 (5.3)	1 (7.1)
Deaths	0 (0.0)	1 (2.1)	0 (0.0)	0 (0.0)
**TEAEs leading to perampanel dose adjustment, n (%)**	49 (69.0)	9 (18.8)	0 (0.0)	0 (0.0)
Withdrawal	8 (11.3)	2 (4.2)	0 (0.0)	0 (0.0)
Dose reduction	40 (56.3)	4 (8.3)	0 (0.0)	0 (0.0)
Dose interruption	19 (26.8)	4 (8.3)	0 (0.0)	0 (0.0)
**Most common TEAEs (reported by ≥10 % of patients, any year), n (%)**				
Dizziness	34 (47.9)	6 (12.5)	0 (0.0)	1 (7.1)
Somnolence	15 (21.1)	1 (2.1)	1 (5.3)	0 (0.0)
Gait disturbance	13 (18.3)	2 (4.2)	0 (0.0)	0 (0.0)
Nasopharyngitis	12 (16.9)	2 (4.2)	1 (5.3)	0 (0.0)
Fall	11 (15.5)	4 (8.3)	3 (15.8)	2 (14.3)
Laceration	4 (5.6)	5 (10.4)	2 (10.5)	0 (0.0)
Bronchitis	3 (4.2)	1 (2.1)	2 (10.5)	0 (0.0)
Ingrown nail	0 (0.0)	0 (0.0)	2 (10.5)	0 (0.0)

TEAEs, treatment-emergent adverse events.

Psychiatric TEAEs were reported in 26.8 % (n = 19/71) of patients during Year 1; of these patients, 47.4 % (n = 9/19) had a prior history of psychiatric conditions. Psychiatric TEAEs occurred in 6.3 % (n = 3/48) of patients during Year 2, and 5.3 % (n = 1/19) of patients during Year 3, while none were reported during Year 4. The most commonly reported psychiatric TEAEs (reported in ≥2 patients) during Year 1 were anger (5.6 % [n = 4/71]), insomnia (4.2 % [n = 3/71]), agitation, confusional state, irritability, mood swings and sleep disorders (2.8 % [n = 2/71], each). Suicidal ideation was reported in 1.4 % of patients (n = 1/71). No single psychiatric TEAE was reported in ≥2 patients during Years 2–4. The most common perampanel doses at which psychiatric TEAEs occurred across all time periods were 8 mg (7.0 % [n = 5/71]) and 12 mg (8.5 % [n = 6/71]).

TEAE incidences in patients aged ≥60 years, stratified by concomitant EIASM use at baseline, are presented in Table S3. During Years 1, 2, and 3, the incidence of TEAEs was similar between patients receiving EIASMs and those receiving non-EIASMs. During Year 1, TEAE incidence was 86.4 % (n = 38/44) in patients receiving EIASMs vs 88.9 % (n = 24/27) in those receiving non-EIASMs. During Year 2, TEAE incidence was 60.6 % (n = 20/33]) in patients receiving EIASMs vs 60.0 % (n = 9/15) in patients receiving non-EIASMs, and during Year 3, it was 46.2 % (n = 6/13) in patients receiving EIASMs vs 50.0 % (n = 3/6) in those receiving non-EIASMs. During Year 4, TEAE incidence was lower in patients receiving EIASMs (50.0 % [n = 5/10]) vs non-EIASMs (75.0 % [n = 3/4]). However, interpretations of data from Years 3 and 4 may be limited by the smaller patient numbers. Serious TEAEs were reported in 33.8 % (n = 24/71) of patients overall; of those, 38.6 % (n = 17/44) of patients received EIASMs and 25.9 % (n = 7/27) of patients received non-EIASMs. During Year 1, the most commonly reported TEAE in patients receiving EIASMs or non-EIASMs was dizziness (EIASMs, 47.7 % [n = 21/44]; non-EIASMs, 48.1 % [n = 13/27]). During Year 2, the most commonly reported TEAE with EIASMs was dizziness (14.7 % [n = 5/34]); with non-EIASMs it was diarrhea (12.5 % [n = 2/16]).

Psychiatric TEAEs occurred in 20.5 % (n = 9/44) and 37.0 % (n = 10/27) of patients in the EIASM and non-EIASM subgroups, respectively, during Year 1; in 5.9 % (n = 2/34) and 6.3 % (n = 1/16) of patients in the EIASM and non-EIASM subgroups, respectively, during Year 2; and in 7.7 % (n = 1/13) of patients who received EIASMs and in none of those who received non-EIASMs during Year 3. No patients reported psychiatric TEAEs during Year 4. The most common psychiatric TEAE during Year 1 (occurring in ≥2 patients across subgroups) in patients who received EIASMs was anger (9.1 % [n = 4/44] vs non-EIASMs 0.0 % [n = 0/27]), and, in those who received non-EIASMs, was insomnia (11.1 % [n = 3/27] vs EIASMs 0.0 % [n = 0/44]). Further, irritability and sleep disorder were not reported in patients treated with EIASMs (0.0 % [n = 0/44], each) but were reported in those treated with non-EIASMs (7.4 % [n = 2/27] each). Suicidal ideation was reported in only one patient who was treated with non-EIASMs (3.7 % [n = 1/27]) during Year 1. No psychiatric TEAEs were reported in ≥2 patients during Years 2–4. The most common perampanel doses at which psychiatric TEAEs occurred during Year 1 were 8 mg (EIASM, 4.5 % [n = 2/44]; non-EIASM, 11.1 % [n = 3/27]) and 12 mg (EIASM, 6.8 % [n = 3/44]; non-EIASM, 11.1 % [n = 3/27]).

## 4 Discussion

Expanding upon the existing data of perampanel use in older adults [9], we sought to assess the extended clinical outcomes of perampanel from two Phase III OLEx studies (Studies 307 and 335 OLEx) in older adult patients with FS (including FBTCS). The results from this post hoc analysis demonstrated that concomitant perampanel had manageable safety and showed good efficacy over a 4-year time period. The findings from this analysis also indicated that treatment with adjunctive perampanel led to reductions in seizure frequency in the older adult population with FS; this improvement was especially evident in the subset of patients with FBTCS, where the 90 % responder rate during Year 1 was >52 %.

Our study supports the findings reported in a subgroup analysis of Study 304 (NCT00699972), Study 305 (NCT00699582), and Study 306 (NCT00700310), which concluded that treatment with perampanel resulted in similar incidences of TEAEs and provided similar efficacy in older adult (aged 65 years and older) and younger adult patients (aged 18 to less than 65 years) [9]. Moreover, a recent real-world observational study conducted in Japan reported that the use of adjunctive perampanel was effective in reducing seizure frequency with no new safety issues in older adult patients who had FS, including FBTCS, or had generalized tonic-clonic seizures [15].

Changes in metabolism associated with aging and impaired drug clearance, along with increased pharmacological sensitivity can complicate treatment regimens in older adults [18]. To reduce the risk of tolerability issues in older adults, it is recommended to use a lower initial dosing of ASMs and a slower titration schedule than those used for younger adults. In addition, ASM doses in older adults may be reduced to half of those normally prescribed to adult patients under 65 years of age [6].

The efficacy of adjunctive perampanel was shown to be more robust when given concomitantly with non-EIASMs vs EIASMs in patients aged ≥12 years, although tolerability was not affected [11]. In our analysis, we explored whether similar findings can be seen in older adult patients. We observed that in Years 1 and 2, reduction in seizure frequency occurred throughout OLEx studies regardless of seizure types or concomitant EIASM use at baseline. However, reductions in Years 3 and 4 were numerically higher in patients receiving adjunctive perampanel concomitantly with EIASMs compared with non-EIASMs. Conversely, in Year 1, numerically higher responder rates were observed in patients who received concomitant non-EIASMs than those treated with EIASMs. Additionally, while the mean modal dose was similar between patients who received concomitant EIASMs and non-EIASMs, the most common modal dose was different; that is, the most common perampanel modal dose in patients treated with concomitant EIASMs was 12 mg/day vs 8 mg/day in those who received non-EIASMs. These findings are in line with previous observations that adjunctive perampanel efficacy and dosing could be influenced by the type of concomitant ASM received [11]. Specifically, the presence of concomitant EIASMs is known to increase the hepatic elimination of perampanel and reduce its plasma levels [19]. Thus, despite the higher most common perampanel modal dose in patients treated with concomitant EIASMs, exposure is likely to be comparable to that in patients who received concomitant non-EIASMs.

The retention rate data demonstrated a considerable decrease in the proportion of patients continuing adjunctive perampanel by the end of Year 4, suggesting both the complexities of long-term epilepsy management in older adults and the need for careful monitoring of older adult patients on perampanel to optimize long-term outcomes. It is important to note, however, that most patients in this post hoc analysis did not discontinue perampanel due to AEs, and less than 20 % discontinued due to inadequate therapeutic effect over the 4-year period.

The safety profile in the older adult population from Studies 307 and 335 OLEx was aligned with the established safety profile of perampanel in the overall population of these studies with no new safety issues identified during the extended treatment [13,14]. The most frequently reported TEAE in patients aged ≥60 years in Year 1 was dizziness (47.9 % [n = 34/71]), which was comparable between patients receiving concomitant EIASMs and those receiving non-EIASMs. Additionally, during Year 1, psychiatric TEAEs occurred in 26.8 % (n = 19/71) of patients aged ≥60 years and were lower in patients receiving concomitant EIASMs (20.5 % [n = 9/44]) compared with patients receiving non-EIASMs (37.0 % [n = 10/27]). The most common psychiatric TEAEs reported during Year 1 in Studies 307 and 335 OLEx older adult population were anger (5.6 % [n = 4/71]) and insomnia (4.2 % [n = 3/71]). Meanwhile, in the overall patient populations in Studies 307 and 335, the most common psychiatric TEAE was irritability (Study 307: 13.9 %; Study 335: 5.1 %) [13,14]. The rate of withdrawal from perampanel treatment due to TEAEs in older adults during the first year was 11.3 %, which was similar or lower than that in the overall populations including adolescents and younger adults (Study 307: 19 %; Study 335: 10.0 %) [13,14]. The safety results from this analysis are comparable with those from a recent sub-analysis of findings from a prospective, observational study of perampanel (Study 402; NCT02033902); the sub-analysis showed TEAEs experienced by older adult patients (aged ≥65 years) with refractory epilepsy were consistent with those experienced by the overall population (aged 12–77 years) and that no new safety signals were detected [20].

Although the aim of current post hoc analysis was to investigate the efficacy of adjunctive perampanel during prolonged treatment, the data at later timepoints likely represents patients who had a positive response and tolerated adjunctive perampanel well, making them more likely to persist with the treatment. Overall, sustained seizure control was observed in older adult patients with FS, with or without FBTCS, treated with adjunctive perampanel. However, in line with prescribing information, patients treated with concomitant EIASMs may need a higher dose of perampanel to experience an efficacy comparable to that seen in patients treated with non-EIASMs only, without a notable difference in safety.

## 5 Limitations

The small sample size at Years 2–4 constrained the interpretation of the results from this period. It is also worth noting that later timepoints might reflect better seizure outcomes compared with Year 1, as it is likely that patients who experienced insufficient therapeutic effect or intolerable side effects would have discontinued after Year 1. Other limitations include the lack of a control arm in both OLEx studies, as well as the post hoc exploratory nature of these analyses. Further long-term analyses in a larger population of older adults are needed.

## 6 Conclusion

Clinical management of epilepsy in older individuals is challenging due to differences in seizure etiologies and the presence of comorbidities, thus limiting treatment options [21]. Older adults also often receive medications for other conditions or other ASMs. These medications may have pharmacokinetic interactions with perampanel that could lead to adjustment in perampanel dose concentrations [12]; measurement of ASM concentrations is often needed to ensure proper treatment delivery [22].

These post hoc analyses provided insight into the impact of adjunctive perampanel use in patients aged ≥60 years, an underrepresented population in clinical studies. The results obtained in these analyses indicate that, for older adult patients with FS, with or without FBTCS, who are considered appropriate candidates for and are able to remain on perampanel, adjunctive perampanel could provide an effective therapeutic option with a safety profile comparable to that in the overall populations. Nevertheless, in line with prescribing information [10,23,24], patients treated with concomitant EIASMs may need a higher perampanel dose to achieve comparable efficacy to that observed in those treated with non-EIASMs only, without a notable difference in safety. These data will help guide clinical decision-making for older adult patients and provide the basis for further study design in this patient population.

## CRediT authorship contribution statement

**Rohit Marawar:** Methodology, Conceptualization. **Ilo E. Leppik:** Methodology, Conceptualization. **Robert T. Wechsler:** Writing – review & editing, Writing – original draft, Supervision, Investigation, Formal analysis, Data curation. **Anna Patten:** Writing – review & editing, Writing – original draft, Formal analysis. **Leock Y. Ngo:** Writing – review & editing, Writing – original draft, Methodology, Investigation, Formal analysis, Conceptualization.

## Declaration of competing interest

The authors declare the following financial interests/personal relationships which may be considered as potential competing interests: Rohit Marawar has received grant support from Eisai; has been a clinical trial investigator for Xenon; has carried out consulting for Jazz Pharmaceuticals; has served on advisory boards for SK Life Science; has received speaker bureau honoraria for Neurelis.

Ilo E. Leppik has no real or apparent conflicts of interest to disclose in relation to this work.

Robert T. Wechsler has been a clinical trial investigator for Aquestive, Cerevel, Eisai, Eliem, Engage Pharmaceuticals, Epalex, Equilibre, Greenwich Biosciences, Jazz Pharmaceuticals, Otsuka, Receptor Neuroscience, SK Life Science, ThirdRock, UCB Pharmaceuticals, Xenon, and Zogenix; has served on advisory boards and/or carried out consulting work for Aquestive, Cerevel, Eisai, Engage Pharmaceuticals, Engrail, Greenwich Biosciences, Jazz Pharmaceuticals, Novella, Otsuka, SK Life Science, and UCB Pharmaceuticals; has received speaker bureau honoraria for Aquestive, Eisai, Jazz Pharmaceuticals, Neurelis, SK Life Science, and UCB Pharmaceuticals; has pay-for-call arrangements with St. Luke’s Health System in Boise, ID; has served as the medical director of the Epilepsy Center at St. Luke’s Health System in Boise, ID, and as the president of the Epilepsy Foundation of Idaho; and is a member of the Epilepsy Study Consortium and the Executive Committee of the Consortium of Private Epilepsy Centers.

Anna Patten is an employee of Eisai Europe Ltd.

Leock Y. Ngo is a former employee of Eisai Inc.
