# Supplementary material for: Long-term efficacy and safety of perampanel in patients aged 60 years and older with focal seizures: Post hoc analysis of phase III open-label extension studies stratified by enzyme-inducing anti-seizure medication use
Source: Epilepsy Behav Rep. 2025 Oct 10;32:100833. doi: 10.1016/j.ebr.2025.100833 (PMC12573446; doi:10.1016/j.ebr.2025.100833)

**Table S1. The disposition of patients aged ≥60 years included in this analysis of the extension studies (Full Analysis Set)**

|  | Placebo  (n=21) | Perampanel  (n=50) | All patients  (N=71) |
| --- | --- | --- | --- |
| Enrolled, n | 21 | 50 | 71 |
| Not treated, n | 0 | 0 | 0 |
| Treated, n (%) | 21 (100.0) | 50 (100.0) | 71 (100.0) |
| Completed extension studies, n (%) | 5 (23.8) | 14 (28.0) | 19 (26.8) |
| Discontinued extension studies, n (%) | 16 (76.2) | 36 (72.0) | 52 (73.2) |
| Primary reason for discontinuation, n (%) |  |  |  |
| Other | 7 (33.3) | 12 (24.0) | 19 (26.8) |
| Inadequate therapeutic effect | 4 (19.0) | 10 (20.0) | 14 (19.7) |
| AE | 2 (9.5) | 6 (12.0) | 8 (11.3) |
| Patient choice | 2 (9.5) | 6 (12.0) | 8 (11.3) |
| Lost to follow-up | 1 (4.8) | 1 (2.0) | 2 (2.8) |
| Withdrawal of consent | 0 | 1 (2.0) | 1 (1.4) |
| Study terminated by sponsor | 0 | 0 | 0 |

AE, adverse event.

**Table S2.** **Perampanel modal dose in patients aged ≥60 years, stratified by concomitant EIASM use recorded at baseline (Safety Analysis Set)**

|  | **EIASMs**  **(n=44)** | **Non-EIASMs**  **(n=27)** | **All patients**  **(N=71)** |
| --- | --- | --- | --- |
| **Mean (SD) modal dose, mg/day** | 8.9 (3.5) | 8.9 (2.6) | 8.9 (3.2) |
| **Modal dose received (mg), n (%)** |  |  |  |
| 2 | 4 (9.1) | 0 | 4 (5.6) |
| 4 | 3 (6.8) | 3 (11.1) | 6 (8.5) |
| 6 | 6 (13.6) | 2 (7.4) | 8 (11.3) |
| 8 | 7 (15.9) | 10 (37.0) | 17 (23.9) |
| 10 | 4 (9.1) | 4 (14.8) | 8 (11.3) |
| 12 | 20 (45.5) | 8 (29.6) | 28 (39.4) |

EIASM, enzyme-inducing anti-seizure medication; mg, milligram; SD, standard deviation.

**Table S3. Overview of safety outcomes over 4 years in patients aged ≥60 years, stratified by concomitant EIASM use recorded at baseline (Safety Analysis Set)**

|  | **Year 1**  **(n=71)** | | **Year 2**  **(n=48)** | | **Year 3**  **(n=19)** | | | **Year 4**  **(n=14)** | |
| --- | --- | --- | --- | --- | --- | --- | --- | --- | --- |
| **Concomitant ASM(s)** | **EIASMs**  **(n=44)** | **Non-EIASMs**  **(n=27)** | **EIASMs**  **(n=33)** | **Non-EIASMs (n=15)** | **EIASMs**  **(n=13)** | **Non-EIASMs**  **(n=6)** | | **EIASMs**  **(n=10)** | **Non-EIASMs**  **(n=4)** |
| **TEAEs, n (%)** | 38 (86.4) | 24 (88.9) | 20 (60.6) | 9 (60.0) | 6 (46.2) | 3 (50.0) | | 5 (50.0) | 3 (75.0) |
| **Treatment-related TEAEs, n (%)** | 37 (84.1) | 23 (85.2) | 12 (36.4) | 3 (20.0) | 3 (23.1) | 1 (16.7) | | 2 (20.0) | 1 (25.0) |
| **Serious TEAEs, n (%)** | 11 (25.0) | 5 (18.5) | 9 (27.3) | 1 (6.7) | 0 (0.0) | 1 (16.7) | | 1 (10.0) | 0 (0.0) |
| Deaths | 0 (0.0) | 0 (0.0) | 1 (3.0) | 0 (0.0) | 0 (0.0) | 0 (0.0) | | 0 (0.0) | 0 (0.0) |
| **TEAEs leading to study drug dose adjustment, n (%)** | | | | | | |  | | |
| Withdrawal | 4 (9.1) | 4 (14.8) | 1 (3.0) | 1 (6.7) | 0 (0.0) | 0 (0.0) | | 0 (0.0) | 0 (0.0) |
| Dose reduction | 26 (59.1) | 14 (51.9) | 3 (9.1) | 1 (6.7) | 0 (0.0) | 0 (0.0) | | 0 (0.0) | 0 (0.0) |
| Dose interruption | 13 (29.5) | 6 (22.2) | 3 (9.1) | 1 (6.7) | 0 (0.0) | 0 (0.0) | | 0 (0.0) | 0 (0.0) |
| **Most common TEAEs (reported by ≥10% of patients in any group, Year 1), n (%)** | | | | | | | | | |
| Dizziness | 21 (47.7) | 13 (48.1) | 5 (15.2) | 1 (6.7) | 0 (0.0) | 0 (0.0) | | 1 (10.0) | 0 (0.0) |
| Somnolence | 9 (20.5) | 6 (22.2) | 1 (3.0) | 0 (0.0) | 0 (0.0) | 1 (16.7) | | 0 (0.0) | 0 (0.0) |
| Nasopharyngitis | 6 (13.6) | 6 (22.2) | 1 (3.0) | 1 (6.7) | 1 (7.7) | 0 (0.0) | | 0 (0.0) | 0 (0.0) |
| Gait disturbance | 6 (13.6) | 7 (25.9) | 2 (6.1) | 0 (0.0) | 0 (0.0) | 0 (0.0) | | 0 (0.0) | 0 (0.0) |
| Headache | 6 (13.6) | 1 (3.7) | 0 (0.0) | 1 (6.7) | 0 (0.0) | 0 (0.0) | | 0 (0.0) | 0 (0.0) |
| Balance disorder | 5 (11.4) | 2 (7.4) | 0 (0.0) | 0 (0.0) | 0 (0.0) | 0 (0.0) | | 0 (0.0) | 0 (0.0) |
| Nausea | 5 (11.1) | 1 (3.7) | 1 (3.0) | 0 (0.0) | 0 (0.0) | 1 (16.7) | | 0 (0.0) | 0 (0.0) |
| Fall | 4 (9.1) | 7 (25.9) | 1 (3.0) | 0 (0.0) | 3 (23.1) | 0 (0.0) | | 1 (10.0) | 1 (25.0) |
| Fatigue | 4 (9.1) | 3 (11.1) | 1 (3.0) | 0 (0.0) | 1 (7.7) | 0 (0.0) | | 0 (0.0) | 0 (0.0) |
| Hypertension | 3 (6.8) | 3 (11.1) | 3 (9.1) | 0 (0.0) | 0 (0.0) | 0 (0.0) | | 0 (0.0) | 0 (0.0) |
| Contusion | 2 (4.5) | 3 (11.1) | 2 (6.1) | 0 (0.0) | 0 (0.0) | 0 (0.0) | | 0 (0.0) | 0 (0.0) |
| Weight increase | 2 (4.5) | 3 (11.1) | 1 (3.0) | 1 (6.7) | 1 (7.7) | 0 (0.0) | | 0 (0.0) | 0 (0.0) |
| Arthralgia | 1 (2.3) | 3 (11.1) | 0 (0.0) | 0 (0.0) | 1 (7.7) | 0 (0.0) | | 0 (0.0) | 0 (0.0) |
| Ataxia | 1 (2.3) | 3 (11.1) | 1 (3.0) | 1 (6.7) | 1 (7.7) | 0 (0.0) | | 0 (0.0) | 0 (0.0) |
| Dry mouth | 1 (2.3) | 3 (11.1) | 0 (0.0) | 0 (0.0) | 0 (0.0) | 0 (0.0) | | 0 (0.0) | 0 (0.0) |
| Dysarthria | 1 (2.3) | 3 (11.1) | 0 (0.0) | 0 (0.0) | 0 (0.0) | 0 (0.0) | | 0 (0.0) | 0 (0.0) |
| Urinary tract infection | 1 (2.3) | 4 (14.8) | 0 (0.0) | 0 (0.0) | 1 (7.7) | 0 (0.0) | | 1 (10.0) | 0 (0.0) |
| Musculoskeletal pain | 0 (0.0) | 3 (11.1) | 1 (3.0) | 0 (0.0) | 0 (0.0) | 0 (0.0) | | 0 (0.0) | 0 (0.0) |
| Insomnia | 0 (0.0) | 3 (11.1) | 0 (0.0) | 0 (0.0) | 0 (0.0) | 0 (0.0) | | 0 (0.0) | 0 (0.0) |

Patients with >1 TEAE(s) with the same preferred term are counted only once for that preferred term.

EIASM, enzyme-inducing anti-seizure medication; TEAE, treatment-emergent adverse event.

**Figure S1. Overview of time to discontinuation over 4 years in patients aged ≥60 years**


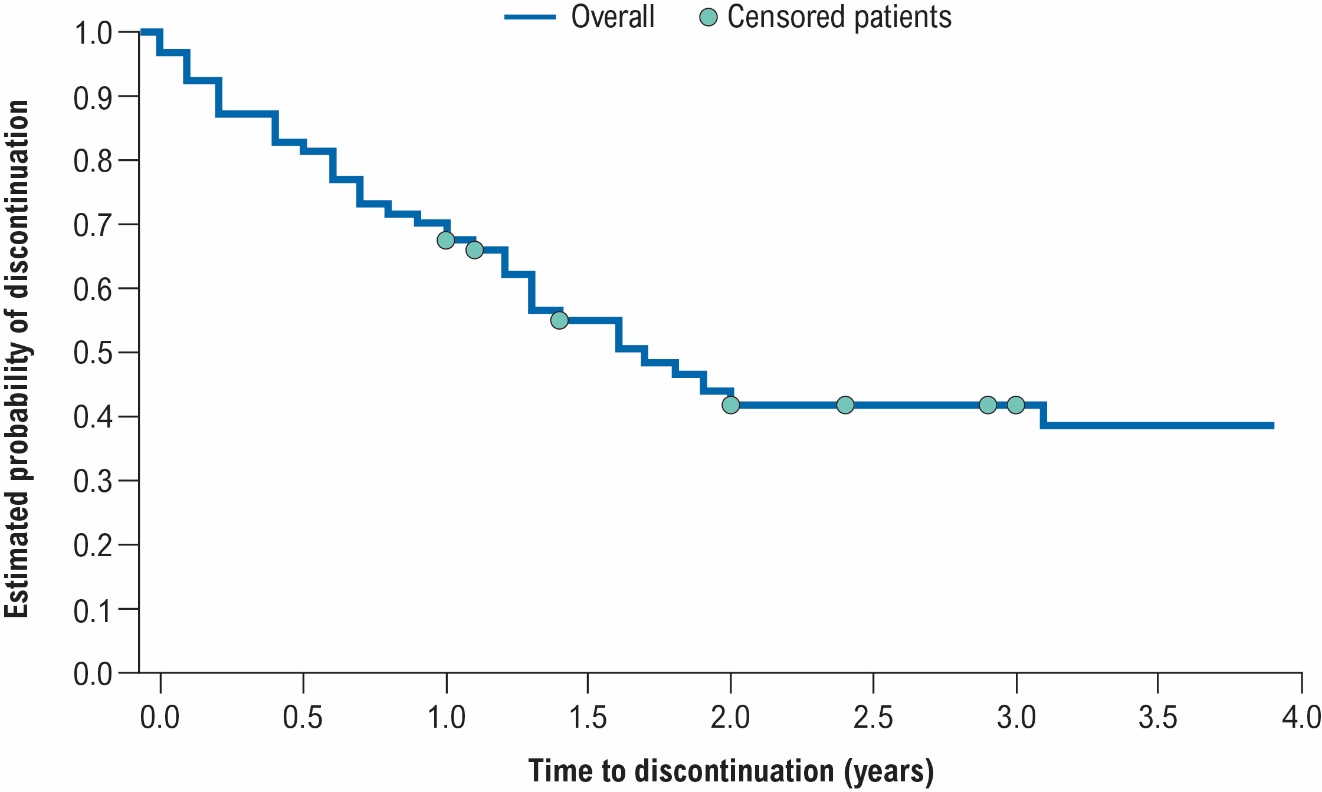

Supplement: Supplementary Data 1 [file mmc1.docx]
